# Supplementary figures and images for: Essential roles for deubiquitination in Leishmania life cycle progression
Source: PLoS Pathog. 2020 Jun 16;16(6):e1008455. doi: 10.1371/journal.ppat.1008455 (PMC7319358; doi:10.1371/journal.ppat.1008455)

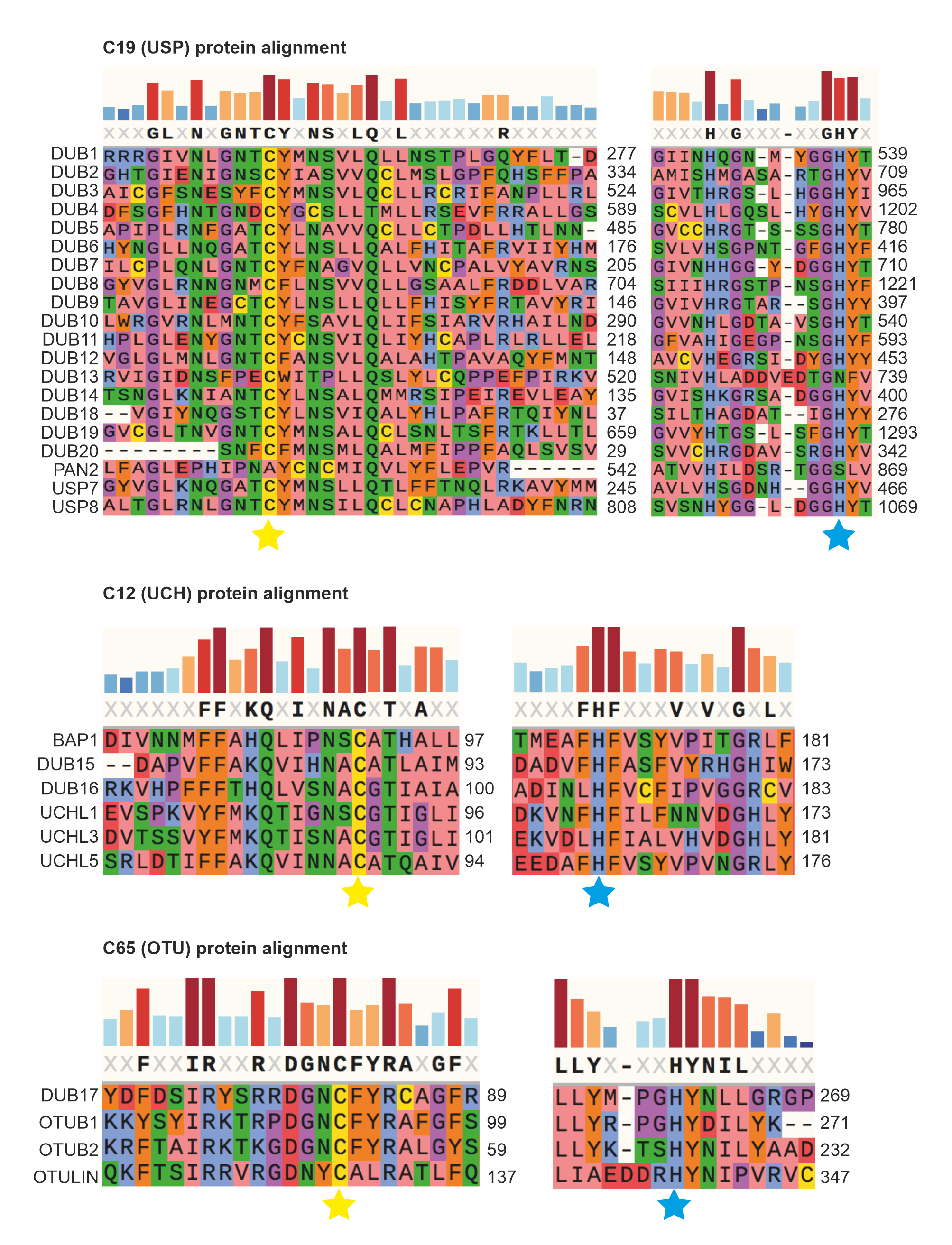

Supplement: S1 Fig — Multiple protein sequence alignment of Leishmania DUBs and selected reference human DUB sequences showing their active residues. Human DUB sequences were obtained from UniProtKB: PAN2 (Q504Q3), USP7 (Q93009), USP8 (P40818), BAP1 (Q92560), UCHL1 (P09936), UCHL3 (P15374), UCHL5 (Q9Y5K5), OTUB1 (Q96FW1), OTUB2 (Q96DC9) and OTULIN (Q96BN8). C19, C12 and C65 DUB families were aligned using the T-Coffee multiple sequence alignment program. A yellow star represents the active cysteine and the blue star represents the active histidine. (TIF) [file ppat.1008455.s001.tif]

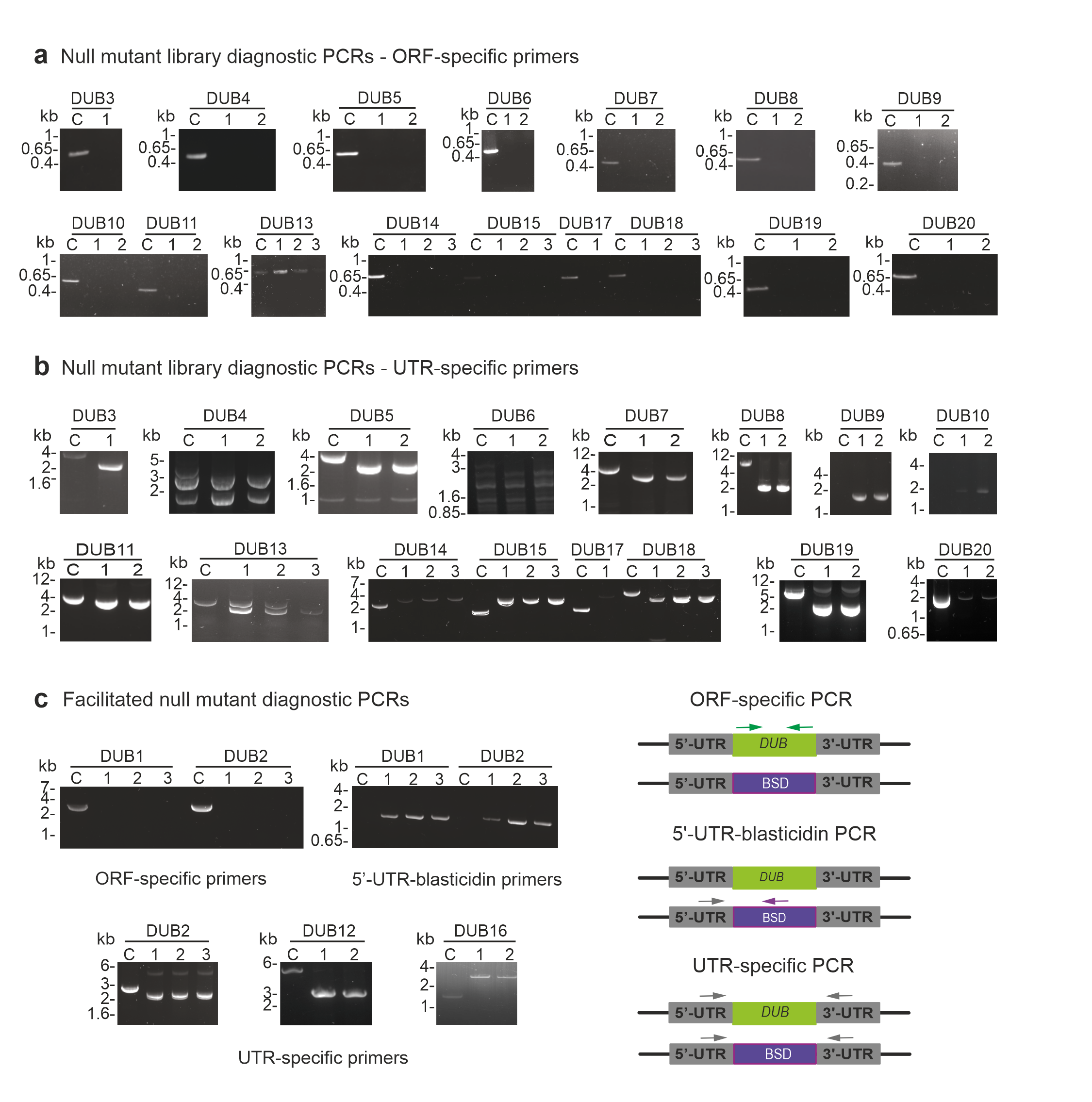

Supplement: S2 Fig — Diagnostic PCRs were performed to check the successful generation of null and facilitated null mutants. PCR analysis of genomic DNA of at least two individual clones (null mutants, CL1, CL2 etc) or two separate populations (facilitated null mutants), using the indicated set of primers: ORF-specific primers (A), UTR-specific primers (B) or ORF-specific, 5’-UTR-blasticidin or UTR-specific primers (C). As a control, the parental Cas9 T7 cell line was used. The resulting amplicons were resolved on a 1% agarose gel and stained with SYBR safe. The expected size of the resulting amplicons is presented in S3 Table. A schematic representation of the wild type, heterozygous, null mutant and facilitated null mutant genomic loci including the diagnostic primers (green arrow primers bind to ORF, grey arrow primers bind to UTRs and amplify across the loci, purple arrow primers binds to the blasticidin resistance marker) is also shown in (C). (TIF) [file ppat.1008455.s002.tif]

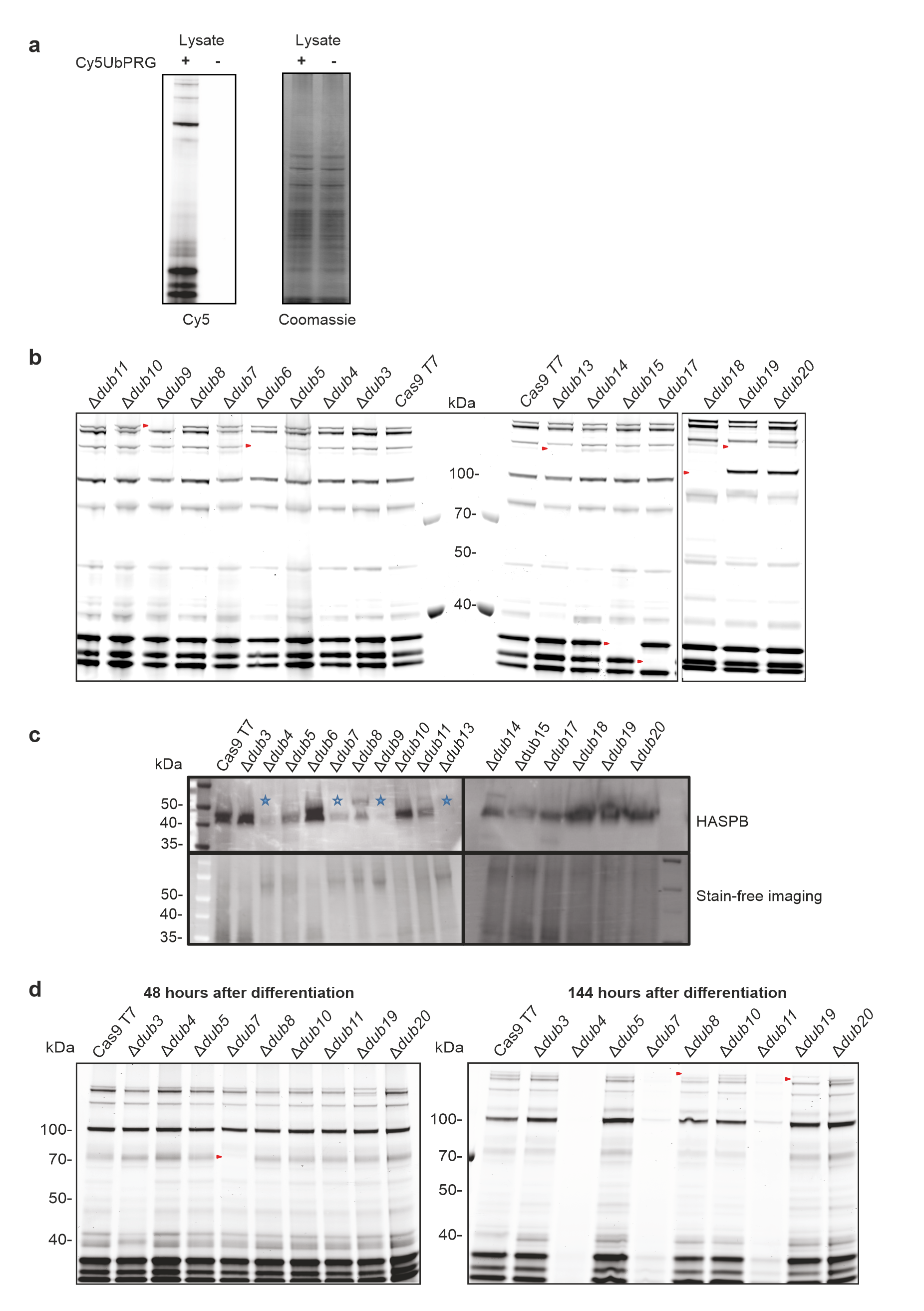

Supplement: S3 Fig — (A) Lysate extracted from log-phase L. mexicana promastigotes treated with or without Cy5UbPRG for 30 min. Proteins were separated by SDS-PAGE and in-gel fluorescence (Cy5) was captured using a Typhoon imager followed by Coomassie staining as a loading control. (B) Lysates extracted from null mutant lines of log-phase L. mexicana promastigotes treated with Cy5UbPRG for 30 min. In-gel fluorescence images were obtained as for (A). The red arrowhead shows the position where an active DUB is missing compared to the parental Cas9 T7 cell line. (C) Western blot analysis of 2 x 10^7^ axenic amastigotes. Samples were separated in a 4–15% protein gel. The stain-free gel used contains trihalo compounds which, in the presence of the UV-light, react with tryptophan residues, producing fluorescence. The gel was activated by 45 sec UV exposure, proteins were transferred to a PVDF membrane and probed with 1:1,500 dilution of anti-HASPB. Finally, as a loading control the total protein was determined using the stain-free property of the gel. (D) Lysate extracted from differentiated promastigotes to axenic amastigotes (48 h and 144 h after initiation of axenic differentiation) treated with or without Cy5UbPRG for 30 min. Protein was separated in an SDS-PAGE gel, and the image was captured using a Typhoon imager and the gel stained with Coomassie. (TIF) [file ppat.1008455.s003.tif]

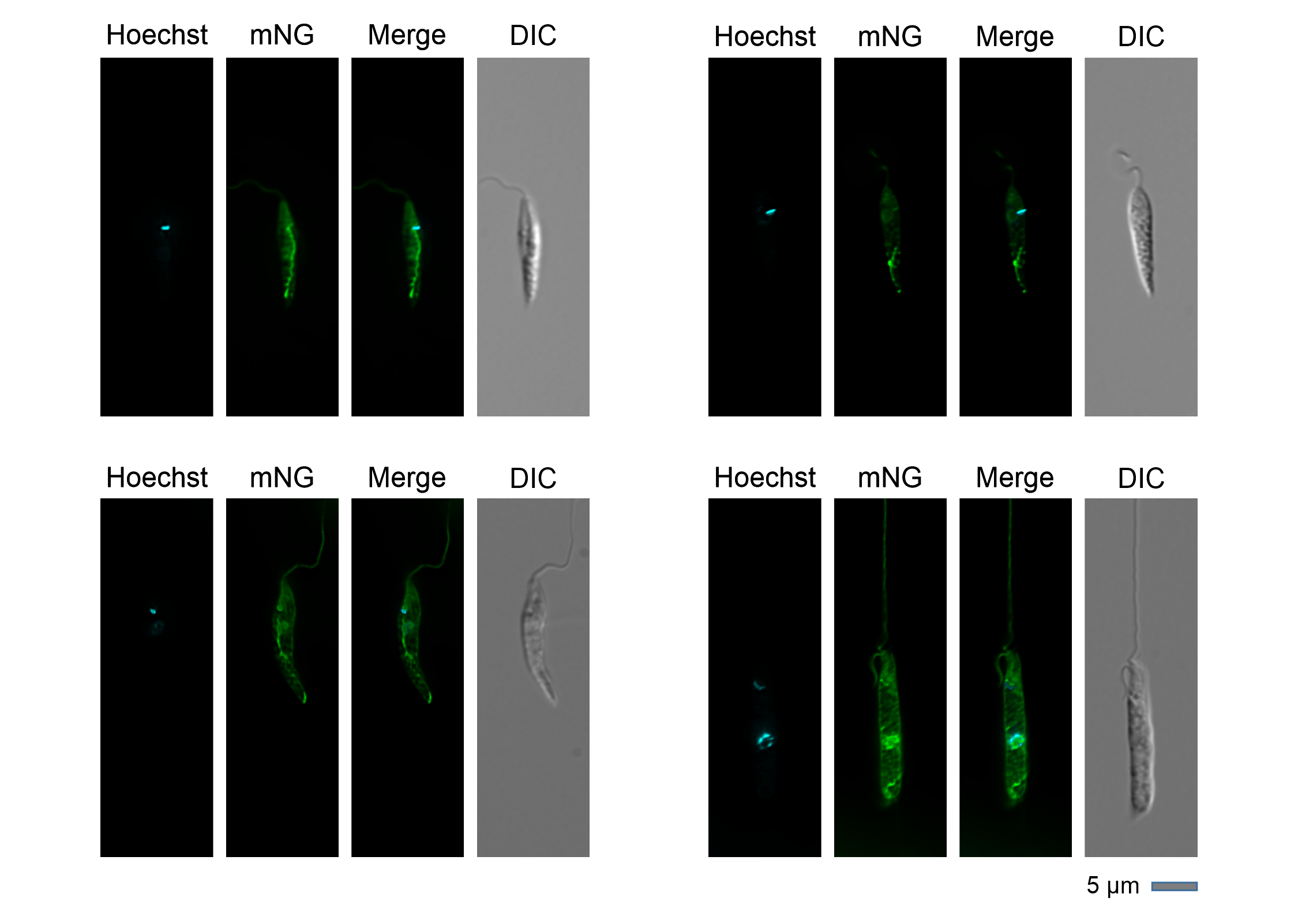

Supplement: S4 Fig — Live cell imaging of L. mexicana procyclic promastigotes expressing mNeonGreen (mNG) tagged DUB2. DNA is stained with Hoechst 33342 and a representative selection of images is shown. DIC, differential interference contrast. (TIF) [file ppat.1008455.s004.tif]

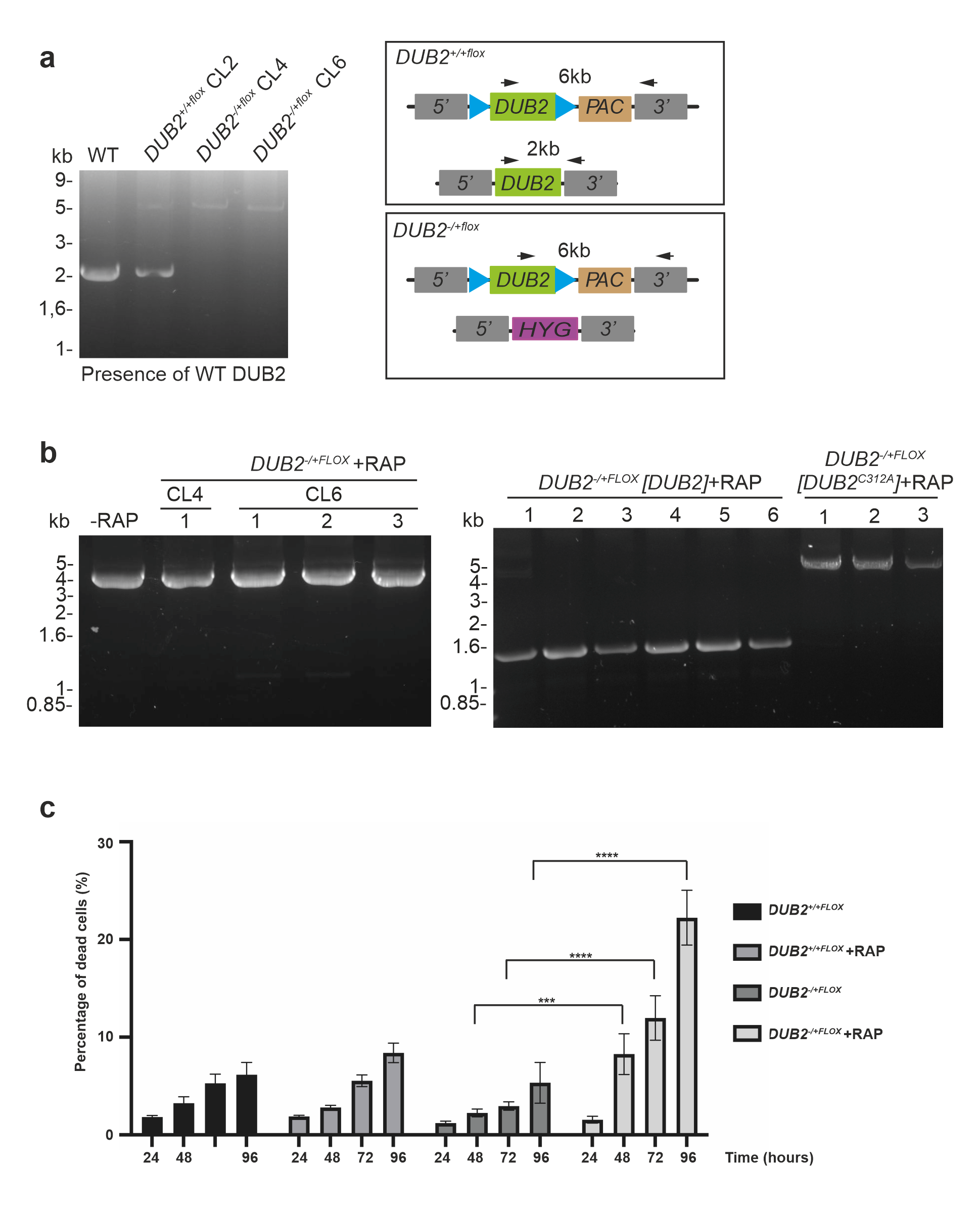

Supplement: S5 Fig — (A) Left: PCR analysis of extracted gDNA demonstrates successful integration of HYG cassette. The replacement of DUB2 wild type allele with the HYG cassette was detected by PCR amplification using the primers shown in the schematic (right). The forward primer was designed to bind to the ORF of DUB2 whereas the reverse primer binds on the 3’ UTR of the target gene. Black arrows represent the primers. (B) PCR amplification of DUB2-/+FLOX Clone 4, 6, DUB2-/+FLOX [DUB2] and DUB2-/+FLOX [DUB2C312A] surviving parasites after treatment with RAP in the clonal assay (Fig 4E). Schematic representation of the primers used for the PCR as well as the expected fragments are shown in Fig 4A. (C) Compiled flow cytometry results of live/dead cells detected using propidium iodide staining in DUB2+/+flox and DUB2-/+flox promastigotes over the course of 96 hours post induction. Cells were grown for 48 hours with or without rapamycin. Afterwards, the cells were seeded at a density of 1 × 10^5^ cells mL−1 and allowed to grow in the presence or absence of 100 nM of RAP. Samples were collected every 24 h. Flow cytometry was used to analyse the samples. The data were then collected and analysed with FlowJo_v10 software, where the percentage of dead cells was determined. The data were analysed in Prism software and an unpaired t-test performed to indicate significance (n = 3) between different time points, comparing treated with RAP to untreated samples. Error bars indicate the standard deviation of a mean. ***p < 0.001, ****p < 0.0001. (TIF) [file ppat.1008455.s005.tif]

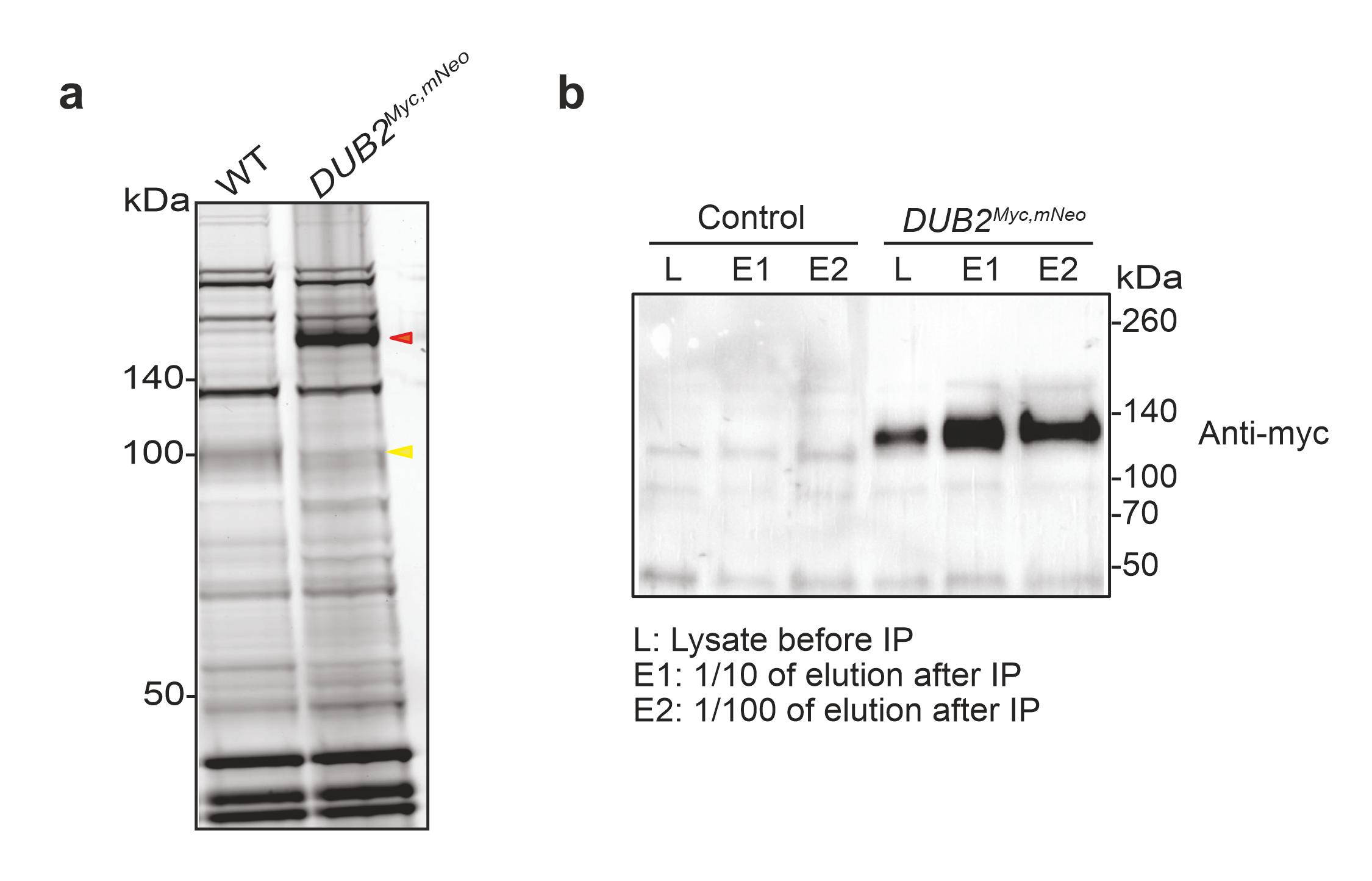

Supplement: S6 Fig — (A) DUB profiling using the ABP Cy5UbPRG. Cell extract from a WT and mNeon:DUB2 was incubated with Cy5UbPRG for 30 min. Proteins were then separated by SDS-PAGE and imaged. The yellow arrow represents the DUB2 WT band whereas the red arrow represents the mNeon-DUB2. (B) Western blotting analysis with anti-myc antibody (1:2,000) of protein cell extract from samples collected after immunoprecipitation of control cell line (WT) and mNeon:DUB2. E1, E2 were samples collected from the elution (myc peptide 0.5 mg mL−1 in PBS). (TIF) [file ppat.1008455.s006.tif]

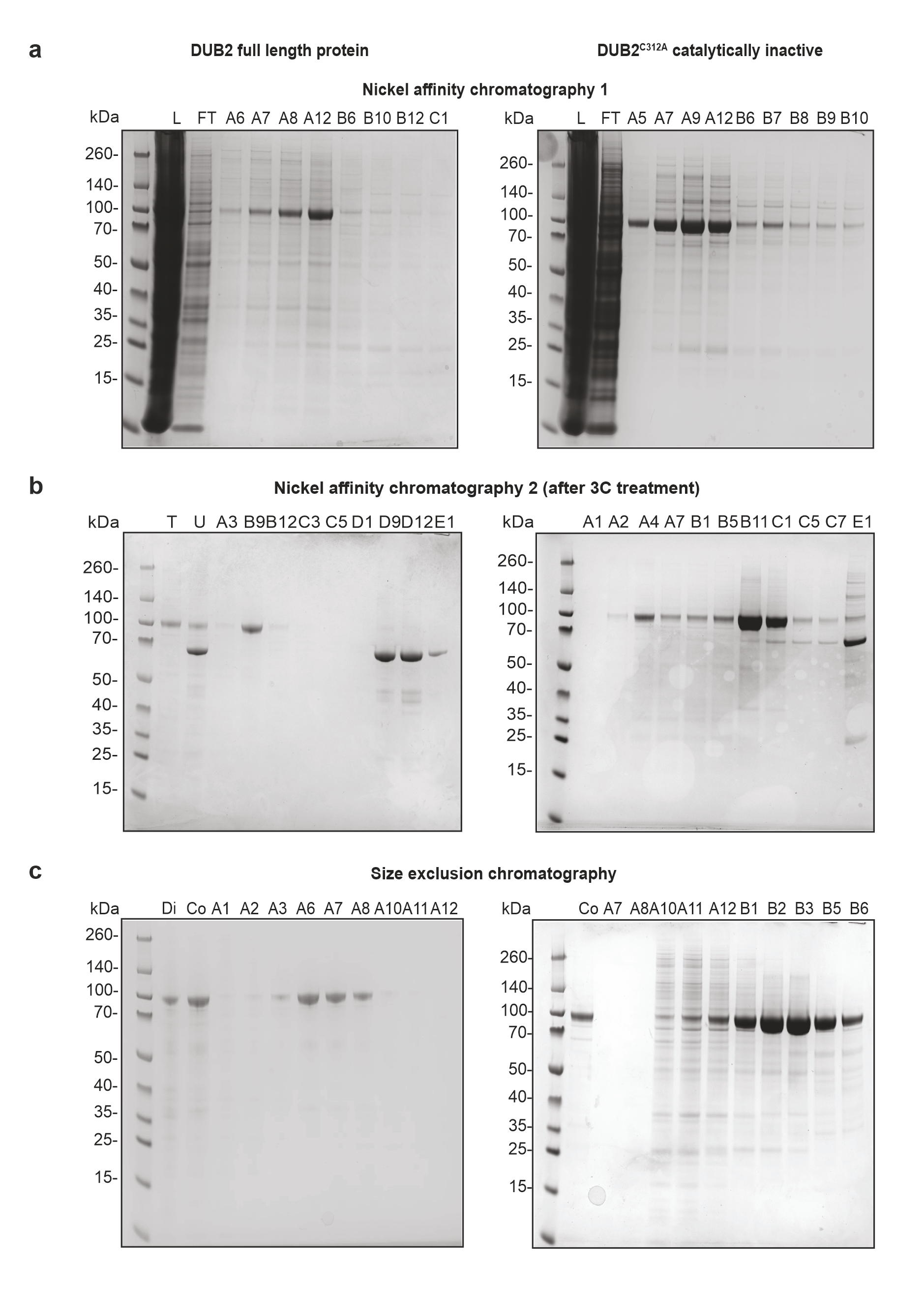

Supplement: S7 Fig — Purification of DUB2 active (left) and DUB2C256 inactive protein (right). (A) Protein gel of the soluble fraction (L), flow though (FT) and eluted fractions across the peak that were collected after Ni2+ affinity chromatography. 10 μL of the protein samples were separated by SDS-PAGE and visualised by InstantBlue Protein Stain. (B) Protein gel of the sample before overnight dialysis with protease 3C for the removal of the his-tag (T), after overnight dialysis (U) and flow through and eluted fractions across the peaks collected after the second Ni2+ affinity chromatography. (C) Protein gel of the diluted (Di) and concentrated (Co) samples before application to a HiLoad 16/600 S75pg column and eluted fractions collected after size-exclusion chromatography. (TIF) [file ppat.1008455.s007.tif]
