# Supplementary material for: Essential roles for deubiquitination in Leishmania life cycle progression
Source: PLoS Pathog. 2020 Jun 16;16(6):e1008455. doi: 10.1371/journal.ppat.1008455 (PMC7319358; doi:10.1371/journal.ppat.1008455)
Supplement: S1 Table — (DOCX) [file ppat.1008455.s008.docx]

Table S1 *Leishmania mexicana* deubiquitinases and ubiquitin-like modifier peptidases

| **DUB Name** | **Merops family** | ***L. mexicana* gene ID** | **Size (kDa)** | ***T. brucei* orthologue** |
| --- | --- | --- | --- | --- |
| DUB1 | C19 | LmxM.27.1270 | 65 | Tb927.11.1930 |
| DUB2 | C19 | LmxM.08_29.2300 | 81 | Tb927.3.4840 |
| DUB3 | C19 | LmxM.29.1200 | 111 | Tb927.6.2690 |
| DUB4 | C19 | LmxM.17.1090 | 140 | Tb927.5.2400 |
| DUB5 | C19 | LmxM.34.2410 | 98 | Tb927.9.13430 |
| DUB6 | C19 | LmxM.34.1740 | 133 | Tb927.9.14470 |
| DUB7 | C19 | LmxM.09.0240 | 105 | Tb927.11.12240 |
| DUB8 | C19 | LmxM.12.0190 | 172 | Tb927.6.1110 |
| DUB9 | C19 | LmxM.15.1300 | 155 | Tb927.9.5520 |
| DUB10 | C19 | LmxM.16.0730 | 200 | Tb927.8.5620 |
| DUB11 | C19 | LmxM.21.0400 | 71 | Tb927.10.2210 |
| DUB12 | C19 | LmxM.24.0620 | 89 | Tb927.11.5270 |
| DUB13 | C19 | LmxM.29.0250 | 126 | Tb927.6.1670 |
| DUB14 | C19 | LmxM.30.0140 | 50 | Tb927.4.3790 |
| DUB15 | C12 | LmxM.24.0420 | 34 | Tb927.11.5140 |
| DUB16 | C12 | LmxM.25.0190 | 25 | Tb9.27.11.250 |
| DUB17 | C65 | LmxM.17.1400 | 30 | Tb927.5.2700 |
| DUB18 | C19 | LmxM.33.4060 | 102 | NONE |
| DUB19 | C19 | LmxM.31.2910 | 148 | Tb927.11.16100 |
| DUB20 | C19 | LmxM.31.1250 | 45 | Tb927.11.14480 |
| DUB21 | C65 | LmxM.16.1385 | 101 | Tb927.8.5050 |
| DUB22 | C65 | LmxM.34.1390 | 74 | Tb927.5.1070 |
| DUB23 | C85 | LmxM.36.6020 | 30 | Tb927.10.8790 |
| UFMP | C78 | LmxM.33.4000 | 56 | Tb927.4.730 |
| PPPDE1 | C97 | LmxM.09.1300 | 24 | Tb927.11.13470 |
| PPPDE2 | C97 | LmxM.09.1310 | 28 | Tb927.11.13470 |
| PPPDE3 | C97 | LmxM.24.0650 | 102 | Tb927.11.5310 |
| PPPDE4 | C97 | LmxM.31.1330 | 70 | Tb927.11.14550 |
| PPPDE5 | C97 | LmxM.32.2260 | 154 | Tb927.9.3400 |
