# Supplementary material for: Essential roles for deubiquitination in Leishmania life cycle progression
Source: PLoS Pathog. 2020 Jun 16;16(6):e1008455. doi: 10.1371/journal.ppat.1008455 (PMC7319358; doi:10.1371/journal.ppat.1008455)
Supplement: S2 Table — (DOCX) [file ppat.1008455.s009.docx]

**Table S2: Proteins specifically affinity-enriched with UbPRG**

| **Accession** | **Description** |
| --- | --- |
| **LmxM.25.0190 (DUB16)** | **C12 family deubiquitinase** |
| **LmxM.17.1400 (DUB17)** | **C65 otubain family deubiquitinase** |
| **LmxM.24.0420 (DUB15)** | **C12 family deubiquitinase** |
| **LmxM.08_29.2300 (DUB2)** | **C19 family deubiquitinase** |
| **LmxM.33.4060 (DUB18)** | **C19 family deubiquitinase** |
| LmxM.17.0710 | Myb-like DNA-binding domain containing protein, putative |
| LmxM.15.1260 | SET domain containing protein, putative |
| LmxM.14.0210 | Domain of unknown function (DUF4201), putative |
| **LmxM.31.2910 (DUB19)** | **C19 family deubiquitinase** |
| LmxM.28.0390 | Nucleus and spindle associated protein 1, putative |
| LmxM.33.3520 | Peroxisome biosynthesis protein-like protein |
| LmxM.36.4060 | Regulator of chromosome condensation 1-like protein |
| LmxM.06.0480 | Hypothetical protein, conserved |
| LmxM.08_29.0850 | High mobility group protein TDP1 |
| LmxM.18.1630 | Hypothetical protein, conserved |
| LmxM.34.3080 | Glycerol kinase, glycosomal, putative |
| LmxM.17.0750 | EF-hand domain pair, putative |
| LmxM.22.0300 | Hypothetical protein, conserved |
| LmxM.31.0640 | Phosphatase, putative |
| LmxM.07.1020 | PIF1 helicase-like protein, putative |
| LmxM.24.1830 | Hypothetical protein, conserved |
| LmxM.19.1520 | Hypothetical protein, conserved |
| LmxM.29.1255 | Hypothetical protein, conserved |
| LmxM.08.1170 | MRB1 complex subunit MRB3010 |
| LmxM.28.1690 | Sulfate transporter-like protein |
| LmxM.17.0815 | G-patch domain containing protein, putative |
| LmxM.27.0220 | Hypothetical protein, conserved |
