# Supplementary material for: Essential roles for deubiquitination in Leishmania life cycle progression
Source: PLoS Pathog. 2020 Jun 16;16(6):e1008455. doi: 10.1371/journal.ppat.1008455 (PMC7319358; doi:10.1371/journal.ppat.1008455)
Supplement: S3 Table — (DOCX) [file ppat.1008455.s010.docx]

Table S3. Oligonucleotides used in this study

| **Oligo No.** |  | **Flank** | **Description** | **Gene ID** | **Sequence** | **Annotations** |
| --- | --- | --- | --- | --- | --- | --- |
| OL4697 | F | 5’ | Amplification of *DUB2* flanking regions for Gateway entry clone generation | LmxM08.29.2300 | GGGGACAACTTTGTATAGAAAAGTTG**TTAATTAA**TACGACGTGGGGAGGCCA | attB4, ***Pac*I** |
| OL4672 | R |  |  |  | GGGGACTGCTTTTTTGTACAAACTTGCGTGCGCGTTCGAGCTAGGG | attB1r |
| OL4673 | F | 3’ |  |  | GGGGACAGCTTTCTTGTACAAAGTGGGACATTTTGACTCTCACGTC | attB2r |
| OL4698 | R |  |  |  | GGGGACAACTTTGTATAATAAAGTTGCG**GTTTAAAC**TTTCCTCCCCAGCACGCACAC | attB3, ***Pme*I** |

| **Oligo No.** |  | **Description** | **Sequence** | **Annotations** |
| --- | --- | --- | --- | --- |
| OL4293 | F | Amplification of *DUB2* to clone into pGL2315 | **GAATTC** ATGCCGTTCCCTGTGACG | ***EcoRI*** |
| OL4294 | R |  | **ACTAGT**TTACGCCTTGCGTTGGAAAAAG | ***SpeI.*** STOP |

| **Oligo No.** |  | **Description** | **Sequence** | **Annotations** |
| --- | --- | --- | --- | --- |
| OL5834 | F | LIC pFast construction for protein expression of LmDUB2 | cagggacccggtCCGTTCCCTGTGACGCAGG | Small letters overlap |
| OL5835 | R |  | cgaggagaagcccggttaTTACGCCTTGCGTTGGAAAA | Small letters overlap |

| **Oligo No.** | **F/R** | **Description** | **Sequence** | **Annotations** |
| --- | --- | --- | --- | --- |
| OL9234 | F | Amplification of pGL1132 backbone for Gipson assembly | ATGGAATTCGCTTGACAAG | Small letters overlap |
| OL9235 | R |  | AGATCTGGTACCATGAGC | Small letters overlap |
| OL7941 | F | Amplification of *DUB2* ORF for Gipson assembly with PGL1132. | gtcaagcgaattccatatgaATGCCGTTCCCTGTGACG | Small letters overlap |
| OL7942 | R |  | cgcccttgctcatggtaccaTTACGCCTTGCGTTGGAAAAAG | Small letters overlap |
| OL7943 | F | Mutagenesis of catalytic cysteine of *DUB2* to Alanine | CGGCAACAGCgcCTACATCGCATC | ***-*** |
| OL7944 | R |  | ATATTCTCGATGCCAGTG | ***-*** |
| OL8479 | F | Amplification of *GT3* ORF for Gipson assembly with PGL1132 | gtcaagcgaattccatatgagatctATGAGCGACAAGTTGGAG | Small letters overlap |
| OL8480 | R |  | cgcccttgctcatggtaccagatctCATTTCTTTCTTCCCGAC | Small letters overlap |
| OL9236 | F | Amplification of *DUB12* ORF for Gipson assembly with PGL1132 | acttgtcaagcgaattccatATGAGCGACAAGTTGGAG | Small letters overlap |
| OL9237 | R |  | ttgctcatggtaccagatctCATTTCTTTCTTCCCGAC | Small letters overlap |
| OL10202 | F | Amplification of *DUB16* ORF for Gipson assembly with PGL1132 | acttgtcaagcgaattccatATGTGGTTCCCGCTGG | Small letters overlap |
| OL10203 | R |  | ttgctcatggtaccagatctTCACTTGTTCACGAGTGCG | Small letters overlap |

| **Oligo No.** | **F/R** | **Gene ID** | **Description** | **Sequence** |
| --- | --- | --- | --- | --- |
| **OL7210** | F | LmxM.27.1270 | Amplification of antibiotic replacement cassette | CTGCCTGCTGTTATCGGAGGCAGAAAAAAGTAATACGACTCACTATAAAACTGGAAGAGCTCAGTCCGGgtataatgcagacctgctgc |
| **OL7211** | R |  |  | CTGCTCAGCATTACAAGGAAGGTAAAAAAGccaatttgagagacctgtgc |
| **OL6496** | F |  | gRNA for 5’ UTR | gaaattaatacgactcactataggAGCTAGATTTGCTCAGGAAGgttttagagctagaaatagc |
| **OL7212** | F |  | gRNA for 3’ UTR | gaaattaatacgactcactataggAGCTATTCAGCGTTGGGGAAgttttagagctagaaatagc |
| **OL6494** | F |  | Amplification of mNeoGreen cassette | CTGCCTGCTGTTATCGGAGGCAGAAAAAAGgtataatgcagacctgctgc |
| **OL6495** | R |  |  | GTAAGAAATCTTATAGTCAAGTTTGAACATactacccgatcctgatccag |
| **OL5613** | F |  | Diagnostic PCR, Primers bind on UTRs | CCTCGTGTTCTGCAAGGA |
| **OL7923** | R |  |  | CTCAGAGGGGATAAAAAGGAG |
| **OL5039** | F |  | Diagnostic PCR, Primers bind on ORF | CGGTTGACTTCACGCAAA |
| **OL 5038** | R |  |  | ACATGTACATAGAGCTAT |
| **OL7213** | F | LmxM.08_29.2300 | Amplification of antibiotic replacement cassette | CCCGTCTTGGCGTGTGTGTGTGTATTACCATAATACGACTCACTATAAAACTGGAAGACTGCCGCGTCCgtataatgcagacctgctgc |
| **OL7214** | R |  |  | CGATGAATTTCACCCACGCACGTGCCAATAccaatttgagagacctgtgc |
| **OL6529** | F |  | gRNA for 5’ UTR | gaaattaatacgactcactataggGTTCGAGCTAGGGGCGCCACgttttagagctagaaatagc |
| **OL7215** | F |  | gRNA for 3’ UTR | gaaattaatacgactcactataggGTGGATTGTACTCGTCGAACgttttagagctagaaatagc |
| **OL6527** | F |  | Amplification of mNeoGreen cassette | CCCGTCTTGGCGTGTGTGTGTGTATTACCAgtataatgcagacctgctgc |
| **OL6528** | R |  |  | ACACGCCTCCTGCGTCACAGGGAACGGCATactacccgatcctgatccag |
| **OL5614** | F |  | Diagnostic PCR, Primers bind on UTRs | GCCGAAACACATTTTCTC |
| **OL7924** | R |  |  | ATACACGCACCGGCGCAG |
| **OL8015** | F |  | Diagnostic PCR, Primers bind on ORF | GAAGGTCACGAGCTCACCAA |
| **OL8016** | R |  |  | TTTGTGCTGCGGGTAGTCAT |
| **OL7216** | F | LmxM.29.1200 | Amplification of antibiotic replacement cassette | CCCTAGGCCGGCTGTCAGGCAGTCCTCCTTTAATACGACTCACTATAAAACTGGAAGTTACTTCTCCGGgtataatgcagacctgctgc |
| **OL7217** | R |  |  | CCCCAGCAAGCAGGGCCCCACTCATGCCCAccaatttgagagacctgtgc |
| **OL6490** | F |  | gRNA for 5’ UTR | gaaattaatacgactcactataggGCTCGAGCCACGGAAGAAAAgttttagagctagaaatagc |
| **OL7209** | F |  | gRNA for 3’ UTR | gaaattaatacgactcactataggCGTCGTGCCCACACCAAGTCgttttagagctagaaatagc |
| **OL6488** | F |  | Amplification of mNeoGreen cassette | CCCTAGGCCGGCTGTCAGGCAGTCCTCCTTgtataatgcagacctgctgc |
| **OL6489** | R |  |  | GTCGACTTCTTCGACGCAGTAGCACGACATactacccgatcctgatccag |
| **OL5615** | F |  | Diagnostic PCR, Primers bind on UTRs | GCGCACTATGTGTCGGCG |
| **OL7925** | R |  |  | GCGTGGTTAGCTTCTGACGC |
| **OL8017** | F |  | Diagnostic PCR, Primers bind on ORF | TGAGCACAACACCCGAATCA |
| **OL8018** | R |  |  | CACTGGAGGAAATGCCGAGT |
| **OL7218** | F | LmxM.17.1090 | Amplification of antibiotic replacement cassette | GGGTGGTGAGGGCCGTACTACTTCCCACCTTAATACGACTCACTATAAAACTGGAAGTGACGCGCGGCCgtataatgcagacctgctgc |
| **OL7219** | R |  |  | CACACGCAGGCTACTCCTACAGCTGCGCCTccaatttgagagacctgtgc |
| **OL6511** | F |  | gRNA for 5’ UTR | gaaattaatacgactcactataggAGGAGGGGAGGGGCGCTCCCgttttagagctagaaatagc |
| **OL7220** | F |  | gRNA for 3’ UTR | gaaattaatacgactcactataggCGGGTCGTTGCATCCGCAAGgttttagagctagaaatagc |
| **OL6509** | F |  | Amplification of mNeoGreen cassette | GGGTGGTGAGGGCCGTACTACTTCCCACCTgtataatgcagacctgctgc |
| **OL6510** | R |  |  | CGCCTCAAGCATACGCCGACCATGACTCATactacccgatcctgatccag |
| **OL5616** | F |  | Diagnostic PCR, Primers bind on UTRs | GCAGCTGCAGGTCACTTC |
| **OL7926** | R |  |  | GGAGAGGCTTGCCGTGC |
| **OL8019** | F |  | Diagnostic PCR, Primers bind on ORF | AAGTGTTTCGTCGTGCGTTG |
| **OL8020** | R |  |  | TACGTGTAAACCTGCCCGTC |
| **OL7221** | F | LmxM.34.2410 | Amplification of antibiotic replacement cassette | TCAGGCTTCATTGTCAGCGAAAGTTAGCCATAATACGACTCACTATAAAACTGGAAGAATGATCTCCGGgtataatgcagacctgctgc |
| **OL7222** | R |  |  | AGACAAGAGATGAAAGACAAGGGGACCGCGccaatttgagagacctgtgc |
| **OL6475** | F |  | gRNA for 5’ UTR | gaaattaatacgactcactataggAATTCTTTCGATTGCGAGCGgttttagagctagaaatagc |
| **OL7206** | F |  | gRNA for 3’ UTR | gaaattaatacgactcactataggGTGACATCCTACCCGTGCAGgttttagagctagaaatagc |
| **OL6473** | F |  | Amplification of mNeoGreen cassette | TCAGGCTTCATTGTCAGCGAAAGTTAGCCAgtataatgcagacctgctgc |
| **OL6474** | R |  |  | CAAGGAAGGGTCGCCGGTAGTGTCCGACATactacccgatcctgatccag |
| **OL5617** | F |  | Diagnostic PCR, Primers bind on UTRs | GCGTGCCTTTGCTGCC |
| **OL7927** | R |  |  | CGAAAAGCACCCCTCACGG |
| **OL8021** | F |  | Diagnostic PCR, Primers bind on ORF | TCGACCGTGTACGACGTTTT |
| **OL8022** | R |  |  | TGTACGGAAACACTACCGCC |
| **OL7223** | F | LmxM.34.1740 | Amplification of antibiotic replacement cassette | ACTCCGTACACATACAAACACGCATATGGGTAATACGACTCACTATAAAACTGGAAGAAGCGTACGTCCgtataatgcagacctgctgc |
| **OL7224** | R |  |  | CGTGTGACCGTGCGGCTAACATGTGAACCGccaatttgagagacctgtgc |
| **OL6472** | F |  | gRNA for 5’ UTR | gaaattaatacgactcactataggCGCAACGCAGAGAAGGAACAgttttagagctagaaatagc |
| **OL7225** | F |  | gRNA for 3’ UTR | gaaattaatacgactcactataggTCTGTTTTGTCGCTTTCATCgttttagagctagaaatagc |
| **OL6470** | F |  | Amplification of mNeoGreen cassette | ACTCCGTACACATACAAACACGCATATGGGgtataatgcagacctgctgc |
| **OL6471** | R |  |  | GTGCTTTGCCACGTCTAAAACTGCAAGCATactacccgatcctgatccag |
| **OL5618** | F |  | Diagnostic PCR, Primers bind on UTRs | CCGTGTCATCATCTTCGCGGCT |
| **OL7928** | R |  |  | AAGCCCTTCGTGTGACCGTGC |
| **OL8023** | F |  | Diagnostic PCR, Primers bind on ORF | AGCTCCTGCAAAACGCAAAG |
| **OL8024** | R |  |  | CGGTGTGTCGAGTTTCCAGA |
| **OL7371** | F | LmxM.09.0240 | Amplification of antibiotic replacement cassette | CTCCTCCTCGGTCCTGTCTCCCTTCGCCCTTAATACGACTCACTATAAAACTGGAAGTATTGTGCAGCCgtataatgcagacctgctgc |
| **OL7373** | R |  |  | ACACAAGGGACTGCAGCAGAGGCTGCCGCTccaatttgagagacctgtgc |
| **OL7372** | F |  | gRNA for 5’ UTR | gaaattaatacgactcactataggGAGAAGGGCAGAGGACGGAAgttttagagctagaaatagc |
| **OL7374** | F |  | gRNA for 3’ UTR | gaaattaatacgactcactataggGTGACCGTCTCTTCCTGCATgttttagagctagaaatagc |
| **OL6521** | F |  | Amplification of mNeoGreen cassette | CTCCTCCTCGGTCCTGTCTCCCTTCGCCCTgtataatgcagacctgctgc |
| **OL6522** | R |  |  | GCGACACCGTGGCAGCAGGCGCAGGCGCATactacccgatcctgatccag |
| **OL7929** | F |  | Diagnostic PCR, Primers bind on UTRs | GTTGGTGTTGTCGACGGC |
| **OL7930** | R |  |  | CAGCAACGGTCTAACCGT |
| **OL8025** | F |  | Diagnostic PCR, Primers bind on ORF | CGGGTGTAACAAGTACCGCT |
| **OL8026** | R |  |  | TTGACAATGCCGTCTAGCGT |
| **OL7375** | F | LmxM.12.0190 | Amplification of antibiotic replacement cassette | GTACACACACACGCAGGGGCACGGACGCCTTAATACGACTCACTATAAAACTGGAAGAGAAACCTTCGGgtataatgcagacctgctgc |
| **OL7377** | R |  |  | CCAACACCCCACCCACCGCACAGACACGTGccaatttgagagacctgtgc |
| **OL7376** | F |  | gRNA for 5’ UTR | gaaattaatacgactcactataggCTCGGTCACCCCTGTTCACGgttttagagctagaaatagc |
| **OL7378** | F |  | gRNA for 3’ UTR | gaaattaatacgactcactataggCACGTGCACCTGCAGCGCATgttttagagctagaaatagc |
| **OL6524** | F |  | Amplification of mNeoGreen cassette | GTACACACACACGCAGGGGCACGGACGCCTgtataatgcagacctgctgc |
| **OL6525** | R |  |  | AGACGCGTGCTTCTGCTCTAGCTGGACCATactacccgatcctgatccag |
| **OL7931** | F |  | Diagnostic PCR, Primers bind on UTRs | AAGGTGTGCGAAGCAGA |
| **OL7932** | R |  |  | ATAACGTAAACGCTGTACG |
| **OL8027** | F |  | Diagnostic PCR, Primers bind on ORF | TCACCACAGAATGGGTCAGC |
| **OL8028** | R |  |  | AGGTCAGAAGCAGAACTGGC |
| **OL7379** | F | LmxM.15.1300 | Amplification of antibiotic replacement cassette | TACCGAGCTGACCCCCTTTTCGCCGTCCCTTAATACGACTCACTATAAAACTGGAAGCGCCATGCATCCgtataatgcagacctgctgc |
| **OL7381** | R |  |  | TGAACAGGATGCCGTGGGGGTGGGGGCGGGccaatttgagagacctgtgc |
| **OL7380** | F |  | gRNA for 5’ UTR | gaaattaatacgactcactataggCGGTGGCTAATCAATGACGAgttttagagctagaaatagc |
| **OL7382** | F |  | gRNA for 3’ UTR | gaaattaatacgactcactataggCGGGAGTGAGAGGAGGGGGTgttttagagctagaaatagc |
| **OL6518** | R |  | Amplification of mNeoGreen cassette | TACCGAGCTGACCCCCTTTTCGCCGTCCCTgtataatgcagacctgctgc |
| **OL6519** | F |  |  | GTACGTATCACCCACATTGGTGGACTCCATactacccgatcctgatccag |
| **OL7933** | F |  | Diagnostic PCR, Primers bind on UTRs | ATTCCTGTTGTCTCCCCAC |
| **OL7934** | R |  |  | AGAGCACAGGAACACCAC |
| **OL8029** | F |  | Diagnostic PCR, Primers bind on ORF | GCCTCGTGTCGCTACCTATC |
| **OL8030** | R |  |  | GTGGACGTGTTTGCTGTGAC |
| **OL7383** | F | \| LmxM.16.0730 \| \| --- \| \| \| \| | Amplification of antibiotic replacement cassette | CCTCCGCACCCGCACCTCTCAGTCTCTCCGTAATACGACTCACTATAAAACTGGAAGTCTTTCCTTCGGgtataatgcagacctgctgc |
| **OL7385** | R |  |  | GCACCAGGCTCCCCATCAGCTGCGGTTCCGccaatttgagagacctgtgc |
| **OL7384** | F |  | gRNA for 5’ UTR | gaaattaatacgactcactataggATGCGCTGTGGGGGCAATCAgttttagagctagaaatagc |
| **OL7386** | F |  | gRNA for 3’ UTR | gaaattaatacgactcactataggAGCATCCCCGAGGTGCACAGgttttagagctagaaatagc |
| **OL6515** | R |  | Amplification of mNeoGreen cassette | CCTCCGCACCCGCACCTCTCAGTCTCTCCGgtataatgcagacctgctgc |
| **OL6516** | F |  |  | GGACGCCGTCCCCGTCACGCAAGCGCTCATactacccgatcctgatccag |
| **OL7935** | F |  | Diagnostic PCR, Primers bind on UTRs | TCCGCTTCTCCCTGTCTT |
| **OL7936** | R |  |  | ATCCTCACGACGCCTCCT |
| **OL8031** | F |  | Diagnostic PCR, Primers bind on ORF | TGTTCGACTTATGGGCCACC |
| **OL8032** | R |  |  | TACGCAGATGCCGATACCAC |
| **OL7387** | F | LmxM.21.0400 | Amplification of antibiotic replacement cassette | GTTCGCGTCACCTCTCCGTATGAGTTGTCGTAATACGACTCACTATAAAACTGGAAGGCGGTTGCATCCgtataatgcagacctgctgc |
| **OL7389** | R |  |  | CGCGCATCGAGCTCGTCTACAGGCTGCCCAccaatttgagagacctgtgc |
| **OL7388** | F |  | gRNA for 5’ UTR | gaaattaatacgactcactataggCTCTTTCTCTCTGCGTTGTTgttttagagctagaaatagc |
| **OL7390** | F |  | gRNA for 3’ UTR | gaaattaatacgactcactataggTAGAGTCGCGTGTGCGCTGGgttttagagctagaaatagc |
| **OL6506** | R |  | Amplification of mNeoGreen cassette | GTTCGCGTCACCTCTCCGTATGAGTTGTCGgtataatgcagacctgctgc |
| **OL6507** | F |  |  | GCCGCGGGACTTGGACGCCTCGTTACCCATactacccgatcctgatccag |
| **OL7937** |  |  | Diagnostic PCR, Primers bind on UTRs | AGTCTCAGACACGGCGAG |
| **OL7938** |  |  |  | GCAGACCCGCCTACACAT |
| **OL8033** |  |  | Diagnostic PCR, Primers bind on ORF | ACTACGGCAACACCTGCTAC |
| **OL8034** |  |  |  | CTTGCTGTGCTTCCAGGAGA |
| **OL7391** | F | LmxM.24.0620 | Amplification of antibiotic replacement cassette | TTCGGCAGCTTCAACGCCACACACGCTGCGTAATACGACTCACTATAAAACTGGAAGATCCCAATTCTTgtataatgcagacctgctgc |
| **OL7393** | R |  |  | TACACACAGGCAGGCAGGCAGGCAGGCCCGccaatttgagagacctgtgc |
| **OL7392** | F |  | gRNA for 5’ UTR | gaaattaatacgactcactataggTGCCGCTCAGCCACCACACAgttttagagctagaaatagc |
| **OL7394** | F |  | gRNA for 3’ UTR | gaaattaatacgactcactataggCCTCTTTGATTGCGCACCTAgttttagagctagaaatagc |
| **OL6500** | F |  | Amplification of mNeoGreen cassette | TTCGGCAGCTTCAACGCCACACACGCTGCGgtataatgcagacctgctgc |
| **OL6501** | R |  |  | CCCGAGCGACCGATTATGCGCGACGTCCATactacccgatcctgatccag |
| **OL7939** |  |  | Diagnostic PCR, Primers bind on UTRs | TCTCTCTTCCTCCCTCCC |
| **OL7940** |  |  |  | CAGTCTCGCACACACACA |
| **OL8035** |  |  | Diagnostic PCR, Primers bind on ORF | CTCTCACAGGTGACGTGGTC |
| **OL8036** |  |  |  | ATGGAACGTCCCTCGTGAAC |
| **OL7395** | F | LmxM.29.0250 | Amplification of antibiotic replacement cassette | GTTACTTTCTTTTCCTTGGCAGCGGTGCCCTAATACGACTCACTATAAAACTGGAAGTCGACCACAGCCgtataatgcagacctgctgc |
| **OL7397** | R |  |  | ACAGGCACTCCACCACGCAGCGGCTCACCAccaatttgagagacctgtgc |
| **OL7396** | F |  | gRNA for 5’ UTR | gaaattaatacgactcactataggCTTTCTACTGTCGGAGCAAGgttttagagctagaaatagc |
| **OL7398** | F |  | gRNA for 3’ UTR | gaaattaatacgactcactataggTCATTTCGTGTTGTGCCCCGgttttagagctagaaatagc |
| **OL6491** | F |  | Amplification of mNeoGreen cassette | GTTACTTTCTTTTCCTTGGCAGCGGTGCCCgtataatgcagacctgctgc |
| **OL6492** | R |  |  | CGGTGTGCTACCTTGCGAACTGAGACTCATactacccgatcctgatccag |
| **OL8690** | F |  | Diagnostic PCR, Primers bind on UTRs | CTTCCACCCACGTACCTTCC |
| **OL8691** | R |  |  | TGTAGAGGGGGTCACGCATA |
| **OL8692** | F |  | Diagnostic PCR, Primers bind on ORF | GTACGCAAAATGTCGGAGGC |
| **OL8693** | R |  |  | CAGCTTCTGAGGCACGGTAA |
| **OL7399** | F | LmxM.30.0140 | Amplification of antibiotic replacement cassette | CCTCCCCCTCCCCTTTACTCGCCCCGTCCCTAATACGACTCACTATAAAACTGGAAGCTCTTGGTTCTTgtataatgcagacctgctgc |
| **OL7401** | R |  |  | GTGAAAGCGCAGGGAAGGAGACGCTTTCCGccaatttgagagacctgtgc |
| **OL7400** | F |  | gRNA for 5’ UTR | gaaattaatacgactcactataggCACGGGCAAATCGTGGCGGAgttttagagctagaaatagc |
| **OL7402** | F |  | gRNA for 3’ UTR | gaaattaatacgactcactataggAGAGTTCTTCGAGGTGCTCGgttttagagctagaaatagc |
| **OL6485** | F |  | Amplification of mNeoGreen cassette | CCTCCCCCTCCCCTTTACTCGCCCCGTCCCgtataatgcagacctgctgc |
| **OL6486** | R |  |  | CTTACCCCACTTGACTTTGATTTCGGCCATactacccgatcctgatccag |
| **OL8694** | F |  | Diagnostic PCR, Primers bind on UTRs | TGGTTGCCTCGATCTTCCAC |
| **OL8695** | R |  |  | GGGCGAATAAACACCACACG |
| **OL8696** | F |  | Diagnostic PCR, Primers bind on ORF | TTCCCTTCACGATGCTCACC |
| **OL8697** | R |  |  | AGCCACGTGTCTGCTTTCTT |
| **OL7403** | F | LmxM.24.0420 | Amplification of antibiotic replacement cassette | CACGCGAAAAGCGAGCGAGACGCAAGACCGTAATACGACTCACTATAAAACTGGAAGAGCTGCACAGCCgtataatgcagacctgctgc |
| **OL7405** | R |  |  | ACCGCTGCCAATGTGACAGCGCAGAATCCAccaatttgagagacctgtgc |
| **OL7404** | F |  | gRNA for 5’ UTR | gaaattaatacgactcactataggCTGTGTGTTTCTGTCGTGATgttttagagctagaaatagc |
| **OL7406** | F |  | gRNA for 3’ UTR | gaaattaatacgactcactataggAAGATACCGTAGATGTGATAgttttagagctagaaatagc |
| **OL6503** | F |  | Amplification of mNeoGreen cassette | CACGCGAAAAGCGAGCGAGACGCAAGACCGgtataatgcagacctgctgc |
| **OL6504** | R |  |  | TAAACACCAATGGCTCTCATTGCTGGGCATactacccgatcctgatccag |
| **OL8698** | F |  | Diagnostic PCR, Primers bind on UTRs | GCTCGCACCGATACGCAAT |
| **OL8699** | R |  |  | AAGAAAGGCAGAGACGCACA |
| **OL8700** | F |  | Diagnostic PCR, Primers bind on ORF | TTAATCGAGAGCGATCCGGC |
| **OL8701** | R |  |  | GCGGTATACAAAGGAGGCGA |
| **OL7407** | F | LmxM.25.0190 | Amplification of antibiotic replacement cassette | CTTCGCCTCACCGTTTTCTGCCGGCTGCCATAATACGACTCACTATAAAACTGGAAGTCTCCGGTTCTTgtataatgcagacctgctgc |
| **OL7409** | R |  |  | GCGCGAGACAGAAAAGAATACAGAAGGTCAccaatttgagagacctgtgc |
| **OL7408** | F |  | gRNA for 5’ UTR | gaaattaatacgactcactataggACAAGGAGAGGCAGCACAAAgttttagagctagaaatagc |
| **OL7410** | F |  | gRNA for 3’ UTR | gaaattaatacgactcactataggAGAATATGGTAATGCGCGCCgttttagagctagaaatagc |
| **OL6497** | F |  | Amplification of mNeoGreen cassette | CTTCGCCTCACCGTTTTCTGCCGGCTGCCAgtataatgcagacctgctgc |
| **OL6498** | R |  |  | CTGCGGATTGCTCTCCAGCGGGAACCACATactacccgatcctgatccag |
| **OL8702** | F |  | Diagnostic PCR, Primers bind on UTRs | TGTGTTCTCCCCCTCTGTTG |
| **OL8703** | R |  |  | CAGGGCCTCAGCACCG |
| **OL8704** | F |  | Diagnostic PCR, Primers bind on ORF | AGCAATCCGCAGGTGATGAA |
| **OL8705** | R |  |  | GAGACTCGTGTCTTCGGCAA |
| **OL7411** | F | LmxM.17.1400 | Amplification of antibiotic replacement cassette | GCAGCGTCGCTGCAGTGCTTCCGCTTTCCGTAATACGACTCACTATAAAACTGGAAGCTAGTCACAGCCgtataatgcagacctgctgc |
| **OL7413** | R |  |  | TGCAGACGACTGTCGCCTTGTTCCCCTCCGccaatttgagagacctgtgc |
| **OL7412** | F |  | gRNA for 5’ UTR | gaaattaatacgactcactataggATTGACGAATTCGCAAGAGAgttttagagctagaaatagc |
| **OL7414** | F |  | gRNA for 3’ UTR | gaaattaatacgactcactataggTATGCACGGATGCAGTCGGTgttttagagctagaaatagc |
| **OL6512** | F |  | Amplification of mNeoGreen cassette | GCAGCGTCGCTGCAGTGCTTCCGCTTTCCGgtataatgcagacctgctgc |
| **OL6513** | R |  |  | GCTCGCGTCGATTGGCAGACCCTCATCCATactacccgatcctgatccag |
| **OL8706** | F |  | Diagnostic PCR, Primers bind on UTRs | TCCAGAGCTTCCCTGCTGTG |
| **OL8707** | R |  |  | TCCAGGAATTTGCTTCGCGT |
| **OL8708** | F |  | Diagnostic PCR, Primers bind on ORF | ATCACGAGAGGAGAGCCTGT |
| **OL8709** | R |  |  | TAGTGGCCGGGCATGTAAAG |
| **OL7415** | F | LmxM.33.4060 | Amplification of antibiotic replacement cassette | ACGCTGTGGATTTTGTCTGCTGGCCCGCCGTAATACGACTCACTATAAAACTGGAAGGAGAAGGTTCTTgtataatgcagacctgctgc |
| **OL7417** | R |  |  | AGCAATACAAGGCGAACATTAACAGAGGATccaatttgagagacctgtgc |
| **OL7416** | F |  | gRNA for 5’ UTR | gaaattaatacgactcactataggTGGGGCGGTCGTGAGGTTGGgttttagagctagaaatagc |
| **OL7418** | F |  | gRNA for 3’ UTR | gaaattaatacgactcactataggGCAAGTGAGCACTGTAGCGTgttttagagctagaaatagc |
| **OL6476** | F |  | Amplification of mNeoGreen cassette | ACGCTGTGGATTTTGTCTGCTGGCCCGCCGgtataatgcagacctgctgc |
| **OL6477** | R |  |  | ATTGTAGATGCCAACGTACCCCGAGCGCATactacccgatcctgatccag |
| **OL8710** | F |  | Diagnostic PCR, Primers bind on UTRs | CGTGCTGCCATCTTATCCCT |
| **OL8711** | R |  |  | ATAAGCCCAAGGAGGGGGTA |
| **OL8712** | F |  | Diagnostic PCR, Primers bind on ORF | ATCACAGCGACGAACCAACT |
| **OL8713** | R |  |  | TAGCCTCCGTTGAACGTCAC |
| **OL7419** | F | LmxM.31.2910 | Amplification of antibiotic replacement cassette | TTCTCTTCAACTCGGGCAACTACATACCCATAATACGACTCACTATAAAACTGGAAGCCGTGGTCATCCgtataatgcagacctgctgc |
| **OL7421** | R |  |  | CTTACCACTCTGCCTCCTCCCCCCGCCCCTccaatttgagagacctgtgc |
| **OL7420** | F |  | gRNA for 5’ UTR | gaaattaatacgactcactataggTGAGAGTGACCACGTGCAATgttttagagctagaaatagc |
| **OL7422** | F |  | gRNA for 3’ UTR | gaaattaatacgactcactataggCCTCTTCCAGTCTCTACTGAgttttagagctagaaatagc |
| **OL6479** | F |  | Amplification of mNeoGreen cassette | TTCTCTTCAACTCGGGCAACTACATACCCAgtataatgcagacctgctgc |
| **OL6480** | R |  |  | CCCAGACCTCAGGGCGCCGTCAGAGCTCATactacccgatcctgatccag |
| **OL8714** | F |  | Diagnostic PCR, Primers bind on UTRs | ACACGAGAGCCCATAAACCC |
| **OL8715** | R |  |  | GACGGTTTGCCCCTTCCTTT |
| **OL8716** | F |  | Diagnostic PCR, Primers bind on ORF | TTCCAGAGGACGAGACGGAT |
| **OL8717** | R |  |  | GGGTGATGTCGCTACCTACG |
| **OL7423** | F | LmxM.31.1250 | Amplification of antibiotic replacement cassette | GTGTATCTCTCTCTTCCTCTGCCGGTACCCTAATACGACTCACTATAAAACTGGAAGTGACCAATTCGGgtataatgcagacctgctgc |
| **OL7425** | R |  |  | ACGCACGCAAGACACAAACCAACCGTCGTCccaatttgagagacctgtgc |
| **OL7424** | F |  | gRNA for 5’ UTR | gaaattaatacgactcactataggACACCAGAAGGGTAGGCTCAgttttagagctagaaatagc |
| **OL7426** | F |  | gRNA for 3’ UTR | gaaattaatacgactcactataggGTTCAAGGTTGGAGGGGGGCgttttagagctagaaatagc |
| **OL6482** | F |  | Amplification of mNeoGreen cassette | GTGTATCTCTCTCTTCCTCTGCCGGTACCCgtataatgcagacctgctgc |
| **OL6483** | R |  |  | CGACACCTGTGACCCCGGTGGCCCACCCATactacccgatcctgatccag |
| **OL8718** | F |  | Diagnostic PCR, Primers bind on UTRs | CAGTGCCAATGGAACAACGG |
| **OL8719** | R |  |  | GCGATGCTTTGTCGAACGTG |
| **OL8720** | F |  | Diagnostic PCR, Primers bind on ORF | CCTTCCAGAAGAAGGGCTGG |
| **OL8721** | R |  |  | TGACATAGTGCCCCCGACTA |

| **pGL No.** | **Gene ID** | **Gene Name** | **Backbone** | **Description** | **Drug Resistance** |
| --- | --- | --- | --- | --- | --- |
| **pNUS-expression plasmids** | | | | | |
| 2762 | LmxM.08_29.2300 | *DUB2* | pGL1132 | pNUS- *DUB2* untagged expression vector | AMPr/ G418r |
| 2763 | LmxM.08_29.2300 | *DUB2* | pGL2762 | pNUS- DUB2 C^707A^ catalytic inactive- untagged expression vector | AMPr/ G418r |
| 2778 | LmxM.36.6280 | *GT3* | pGL1132 | pNUS- *GT3* untagged expression vector | AMPr/ G418r |
| 2784 | LmxM.24.0620 | *DUB12* | PGL1132 | pNUS- DUB12 untagged expression vector | AMPr/ G418r |
|  | LmxM25.0190 | *DUB16* | pGL1132 | pNUS- DUB16 untagged expression vector | AMPr/ G418r |
| **Gateway entry plasmids** | | | | | |
| 2315 | N/A | *loxP-C-GFP* | pDONR221 | LoxP (empty) expression cassette: c-terminal GFP tag | KANr |
| 2411 | LmxM.08_29.2300 | *3’ DUB2 flank* | PDONR P2r-P3 | 3’ Flank (1000bp) ready for Gateway recombination | KANr |
| 2412 | LmxM.08_29.2300 | *5’ DUB2 flank* | pDONR P41-Pr | 5’ Flank (100bp) ready for Gateway recombination | KANr |
| 2727 | LmxM.08_29.2300 | *LoxP-DUB2* | pGL2315 | LoxP *DUB2* expression cassette: untagged | KANr |
| **Gateway expression plasmids** | | | | | |
| 2399 | LmxM.27.1270 | *RIB* | pDEST R4-R3 | *diCre* with 5’ and 3’ 1000bp *homologous flanks for pRIB integration* | AMPr/ BLAr |
| 2727 | LmxM.08_29.2300 | *DUB2* | pDEST R4-R3 | Hygromycin resistance cassette with 1000bp *DUB2* flanks | AMPr/ HYGr |
| 2728 | LmxM.08_29.2300 | *DUB2* | pDEST R4-R3 | *DUB2^flox^ cassette flanked with DUB2 homologous arms* | AMPr/ BLAr |
| **Recombinant protein expression plasmids** | | | | | |
| 2404 | N/A | *-* | pFastBacNKI-his-3C-LIC | Plasmid to clone gene for baculovirus protein expression | AMPr |
| 2689 | LmxM.08_29.2300 | *DUB2* | pFastBacNKI-his-3C-LIC | pFast plasmid with *DUB2* gene | AMPr |
| 2764 | LmxM.08_29.2300 | *DUB2* | pFastBacNKI-his-3C-LIC | pFast plasmid with *DUB2* catalytic inactive gene (DUB2 C^707A^) | AMPr |

| **Gene** | **ORF set of primers (bp)** |  |  | **UTR set of primers (bp)** |  |  |
| --- | --- | --- | --- | --- | --- | --- |
|  | **T7Cas9** | **Het (-/+)** | **Null mutant (-/-)** | **T7Cas9** | **Het (-/+)** | **Null mutant (-/-)** |
| DUB1 | 1132 | 1132 | - | 2000 | 2000, 1998 | 1998 |
| DUB2 | 555 | 555 | - | 2518 | 2518, 1994 | 1994 |
| DUB3 | 482 | 482 | - | 3441 | 3441, 2143 | 2143 |
| DUB4 | 577 | 577 | - | 4191 | 4191, 1973 | 1973 |
| DUB5 | 412 | 412 | - | 3226 | 3226, 2080 | 2080 |
| DUB6 | 518 | 518 | - | 3854 | 3854, 2083 | 2083 |
| DUB7 | 417 | 417 | - | 3038 | 3038, 1911 | 1911 |
| DUB8 | 558 | 558 | - | 5197 | 5197, 2015 | 2015 |
| DUB9 | 422 | 422 | - | 4481 | 4481, 1953 | 1953 |
| DUB10 | 538 | 538 | - | 5839 | 5839, 1868 | 1868 |
| DUB11 | 437 | 437 | - | 2348 | 2348, 2129 | 2129 |
| DUB12 | 537 | 537 | - | 2600 | 2600, 1893 | 1893 |
| DUB13 | 465 | 465 | - | 4137 | 4137, 2476 | 2476 |
| DUB14 | 527 | 527 | - | 1953 | 1953, 2305 | 2305 |
| DUB15 | 477 | 477 | - | 1318 | 1318, 2117 | 2117 |
| DUB16 | 420 | 420 | - | 1310 | 1310, 2321 | 2321 |
| DUB17 | 459 | 459 | - | 1408 | 1408, 2318 | 2318 |
| DUB18 | 476 | 476 | - | 3165 | 3165, 2169 | 2169 |
| DUB19 | 408 | 408 | - | 4420 | 4420, 2120 | 2120 |
| DUB20 | 528 | 528 | - | 1878 | 1878, 2071 | 2071 |
